# Data on liquid gated CNT network FETs on flexible substrates

**DOI:** 10.1016/j.dib.2018.09.093

**Published:** 2018-10-02

**Authors:** Murugathas Thanihaichelvan, Leo A. Browning, Marissa P. Dierkes, Roger Martinez Reyes, Andrew V. Kralicek, Colm Carraher, Colleen A. Marlow, Natalie O.V. Plank

**Affiliations:** a School of Chemical and Physical Sciences, Victoria University of Wellington, Wellington 6021, New Zealand; b The MacDiarmid Institute for Advanced Materials and Nanotechnology, New Zealand; c Department of Physics, University of Jaffna, Jaffna 40000, Sri Lanka; d Physics Department, California Polytechnic State University, San Luis Obispo, CA 93407, United States; e The New Zealand Institute for Plant & Food Research Limited, Auckland 1142, New Zealand

## Abstract

This article presents the raw and analyzed data from a set of experiments performed to study the role of junctions on the electrostatic gating of carbon nanotube (CNT) network field effect transistor (FET) aptasensors. It consists of the raw data used for the calculation of junction and bundle densities and describes the calculation of metallic content of the bundles. In addition, the data set consists of the electrical measurement data in a liquid gated environment for 119 different devices with four different CNT densities and summarizes their electrical properties. The data presented in this article are related to research article titled “*Metallic-semiconducting junctions create sensing hot-spots in carbon nanotube FET aptasensors near percolation”* (doi:10.1016/j.bios.2018.09.021) [1].

**Specifications table**

**Table:** 

Subject area	*Physics*
More specific subject area	*CNT FETs, Electrical properties*
Type of data	*Table, figure*
How data was acquired	*Atomic force microscope (Nanosurf, NaioAFM), Agilent 4156C parameter analyser and Rucker and Kolls probestation.*
Data format	*Raw and analyzed data*
Experimental factors	*CNT film morphology and electrical properties of CNT network FETs*
Experimental features	*The CNT FETs were fabricated by using simple solution processing methods and photolithography. The CNT film morphology was studied using AFM and the electrical characterizations were measured using Agilent 4156C parameter analyser and a Rucker and Kolls probestation.*
Data source location	School of Chemical and Physical Sciences, Victoria University of Wellington, Wellington 6021, New Zealand.
Data accessibility	*All data are available within the paper*
Related research article	*M. Thanihaichelvan, L.A. Browning, M.P. Dierkes, R.M. Reyes, A. V. Kralicek, C. Carraher, C.A. Marlow, N.O. V. Plank, Metallic-semiconducting junctions create sensing hot-spots in carbon nanotube FET aptasensors near percolation, Biosensors and Bioelectronics (2018).*doi:10.1016/J.BIOS.2018.09.021.

**Value of the data**

• A simple method to estimate the metallic content of CNT bundles is provided
• The electrical data on 119 working devices with their electrical properties is included
• Data on transfer curves, and gate leakage of liquid gated network CNT FETs with encapsulated electrodes is presented.

## 1 Data

### 1.1 AFM characterization of the CNT network films

CNT network morphology was characterized for different CNT deposition times using AFM images. The relevant data was extracted from 5 µm × 5 µm AFM images for three samples per deposition time.

#### 1.1.1 CNT bundle diameter distribution

**Fig. 1:**
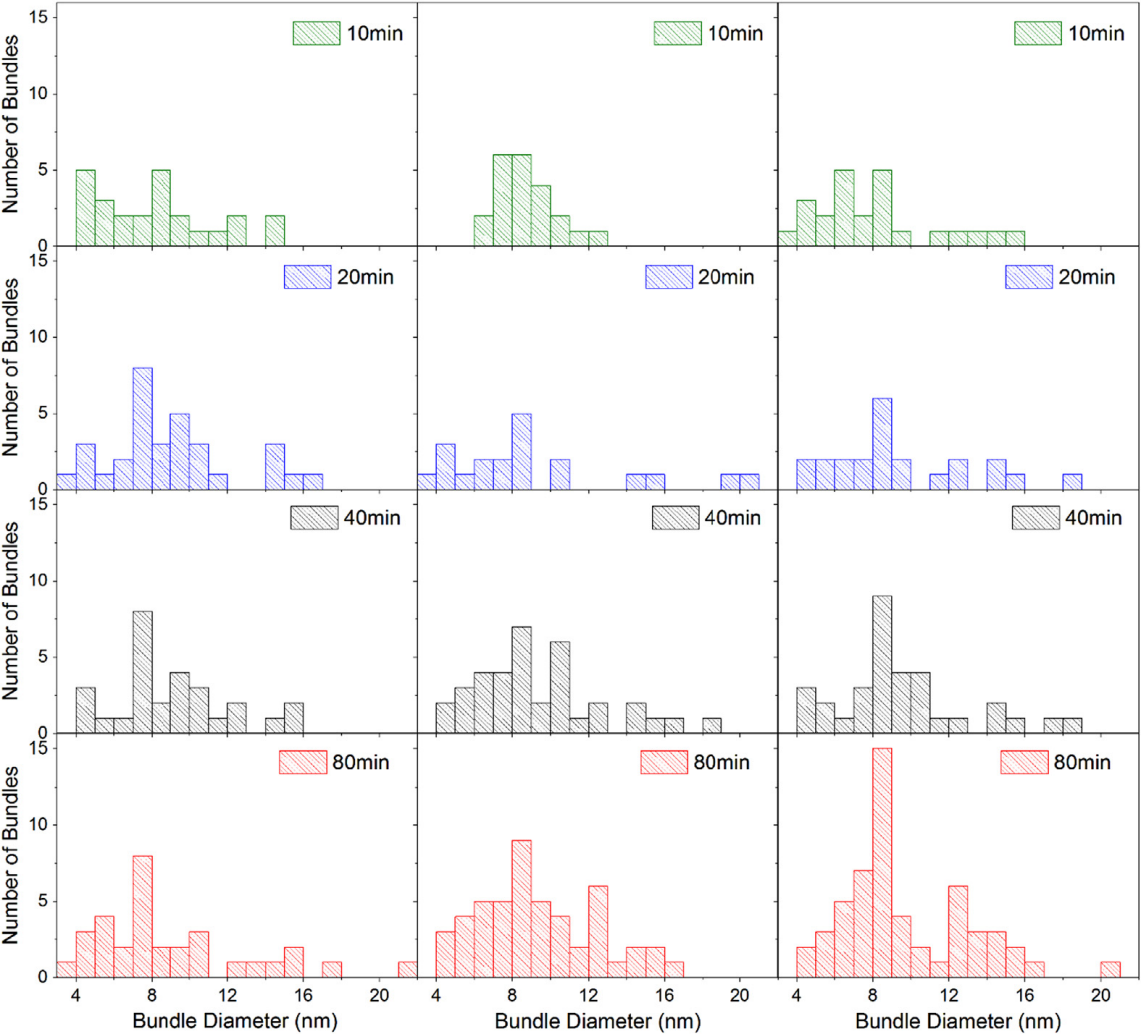
Distribution of bundle diameters across three samples per deposition time, data was collected from 5 µm ×5 µm AFM images.

Bundle heights were found by analysing the AFM scan data using a linear cross section taken perpendicular to each bundle. The height of the bundle was determined by subtracting the surrounding substrate height for that cross section from the bundle height. This value was then determined to be the bundle diameter. Fig. 1 shows the distribution of the number of counts of bundles at a particular diameter across our device films. Specifications for the CNT buckypaper used to create the films are given by the manufacturer (NanoIntegris) state that the CNT diameters are in the range of 1.2 nm to 1.7 nm with the composition of 1% metallic tubes. Modelling a bundle as a cylinder and assuming 2D packing of tubes we then estimate the number of tubes in each bundle, *n*, assuming an average CNT diameter of 1.5 nm [2]. Table 1 lists the *n* values estimated for each bundle diameter observed.

**Table 1:** Bundle diameter and the number of tubes for a given bundle determined from circular tube packing [2].

**Bundle diameter (nm)**	**3**	**4**	**5**	**6**	**7**	**8**	**9**	**10**	**11**	**12**	**13**	**14**	**15**	**16**	**17**	**18**	**19**	**20**	**21**
***n=*****Number of tubes/bundle**	2	5	8	11	16	21	27	34	41	50	58	69	79	89	103	117	131	145	163

We characterize a bundle as metallic if it contains at least one metallic CNT. Since the CNT buckypaper source contained 1% metallic tubes the probability that a given bundle composed of *n* tubes can be characterized as a metallic bundle, is

$$p_{\mathrm{metallic}}\left(n\right)=1-0.99^{n}$$

From this, the percentage of metallic tubes *P*_*metallic*_ in a sample can be determined by

$$P_{\mathrm{metallic}}=\frac{100}{{\sum N}_{n}}\sum_{n=1}^{n=\max}N_{n}p_{\mathrm{metallic}}(n)$$

where *N*_*n*_ is the number of bundles containing *n* tubes taken from the observed distribution of bundle diameters for our devices shown in Fig. 1. *P*_*metallic*_ was calculated for each sample and was averaged across samples of the same deposition time. The average percentage of bundles that were metallic was found to be 22.90 (±0.69)%, 24.28 (±0.64)%, 24.53 (±0.61)%, and 24.66 (±1.44)% for the 10, 20, 40 and 80 min deposited films respectively. The uncertainty results from the variation across samples with the same deposition times. When averaged across all deposition times we find the percentage of metallic bundles in the network to be 24.09 (±0.85)% across all samples.

### 1.2 CNT bundle length distribution

**Fig. 2:**
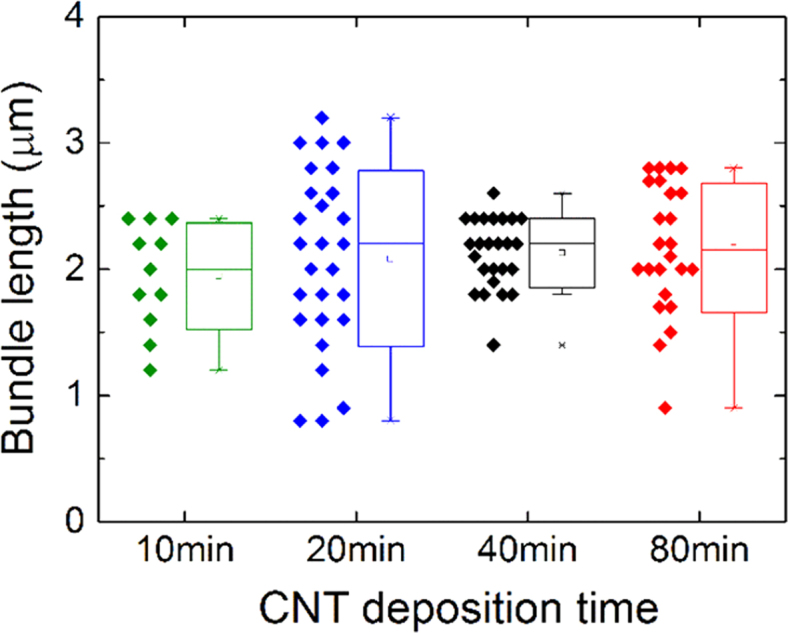
The distribution of CNT bundle length with CNT deposition time. The box indicates the standard deviation and the whiskers indicate the outliers on all graphs.

**Fig. 3:**
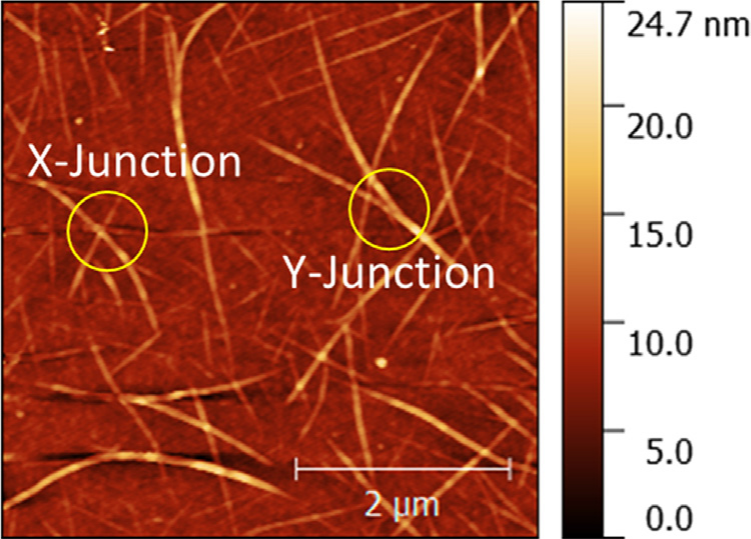
AFM image of a 20 min deposited CNT network on SiO_2_/Silicon substrate showing an example of X and Y type bundle-bundle junctions.

The CNT bundle lengths measured by AFM are shown in Fig. 2 and do not significantly vary with the CNT deposition time. The average bundle lengths are found to be 2.00 (±0.42) µm, 2.09 (±0.63) µm, 2.16 (±0.24) µm, and 2.17 (±0.51) µm for 10, 20, 40, and 80 min deposited films respectively with the uncertainty resulting from the standard deviation of bundle lengths as shown in Fig. 3. The overall average bundle length was calculated to be 2.14 ± 0.45 µm by averaging the bundle length calculated per deposition time.

### 1.3 CNT bundle-bundle junction and bundle density

Fig. 3 shows a typical AFM image of a CNT network with X type and Y type junctions clearly indicated on the figure. A study of junction resistance using conductive AFM measurements by Nirmalraj et al. and Sun et al. reported that Y type junctions where bundles are in close contact alongside one another show little or no resistance change across them, while X type junctions have an abrupt resistance change [3], [4]. Therefore, in our AFM images we identified X and Y type junctions and excluded Y junctions from our count of junction density since only X type junctions contribute significantly to the overall network resistance.

**Fig. 4:**
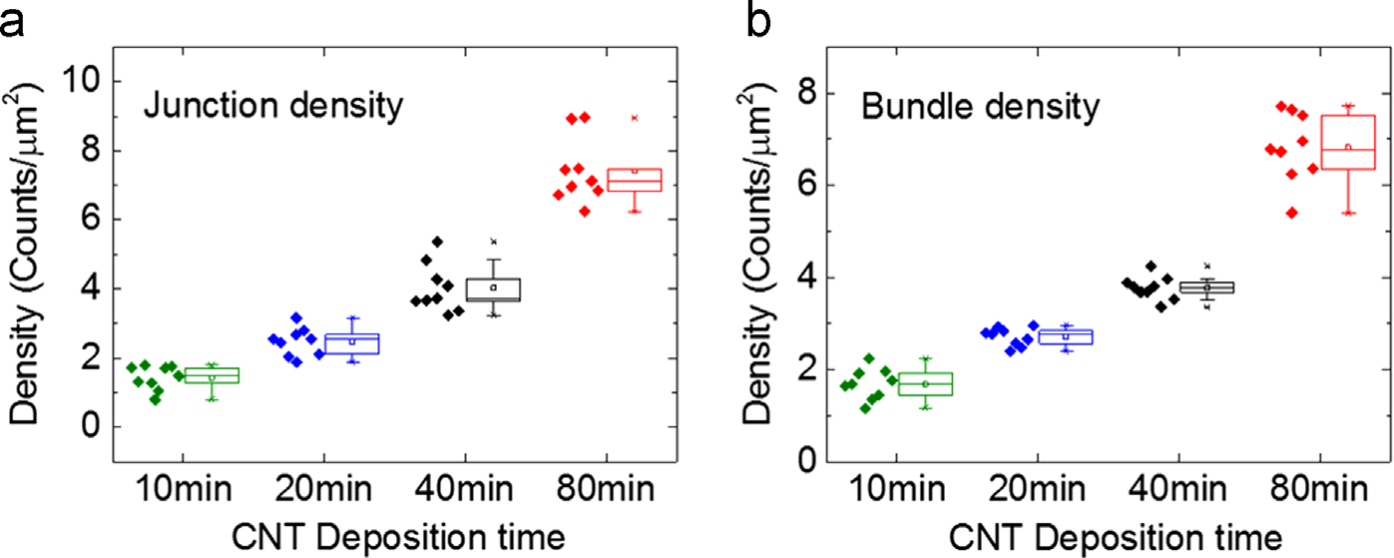
The distribution of (a) bundle-bundle junction density and (b) the density of bundles across chips with the same fabrication time. Data-points are the density calculated from nine 5 µm × 5 µm AFM images taken across 3 chips with the same fabrication time. The box indicates the standard deviation and the whiskers indicate the outliers on all graphs.

The junction and total bundle density values reported in the main text in Fig. 3 are taken from averaging across multiple chips of the same deposition time. The raw data along with the mean and standard deviation per CNT deposition time is shown in Fig. 4.

### 1.4 Electrical characterization of liquid gated CNT FETs and aptasensors

**Fig. 5:**
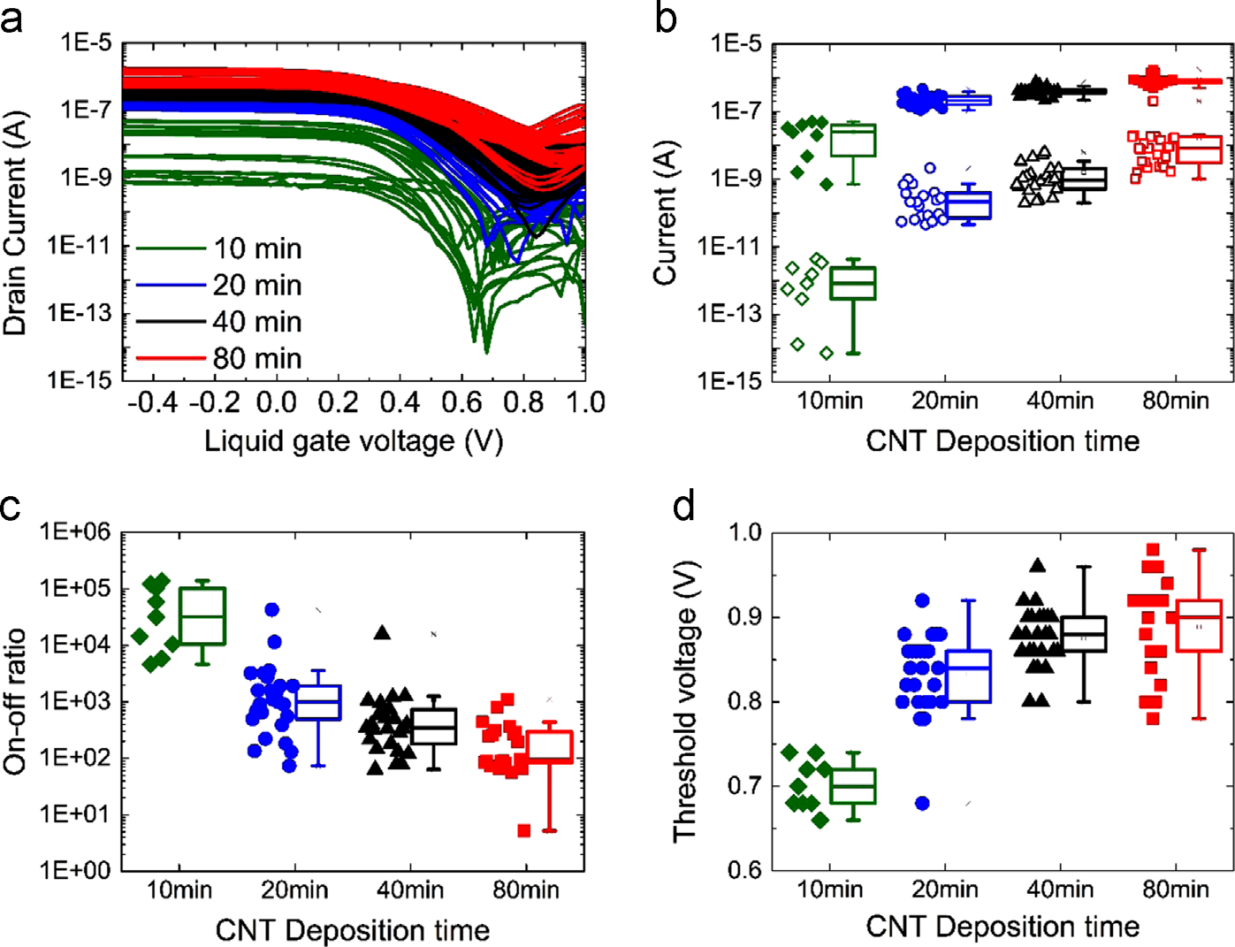
(a) The transfer (*I*_*ds*_–*V*_*lg*_) characteristics of the CNT FETs fabricated with different CNT deposition times under liquid gated conditions, distribution of (b) On (filled shapes) and Off (hollow shapes) currents, (c) on-off ratios and (d) threshold voltages of the 119 functional CNT FETs with different deposition times, the boxes indicate the 25^th^ and 75^th^ percentile and the whiskers indicate the outliers on all graphs.

**Fig. 6:**
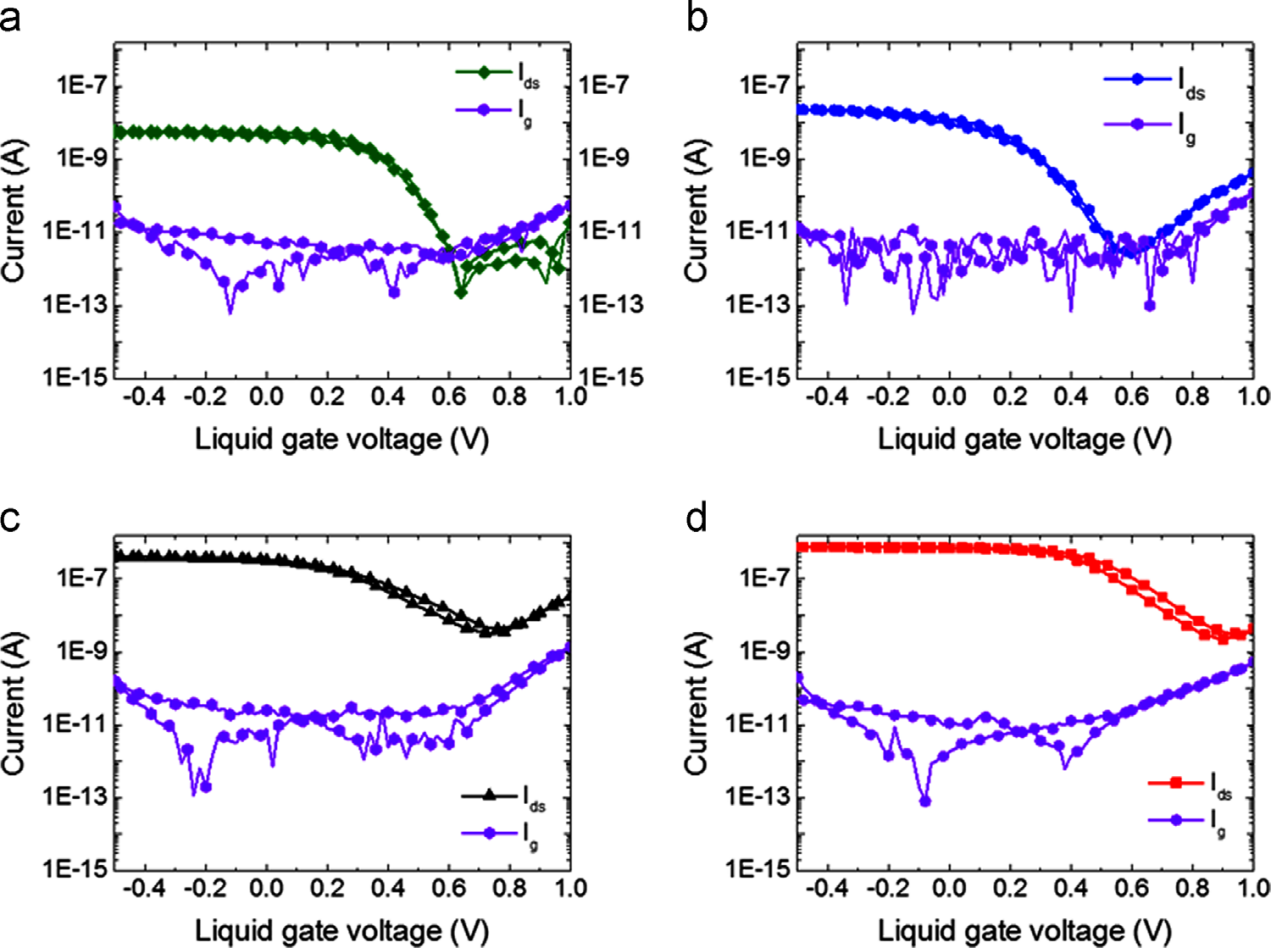
Transfer curves and gate currents of the devices under liquid gated conditions at *V*_*ds*_=100 mV, fabricated with CNT films on kapton substrate with deposition times of (a) 10, (b) 20, (c) 40 and (d) 80 min.

Fig. 5 shows the drain source current *I*_*ds*_ with the liquid gate voltage *V*_*lg*_. The gate currents for CNT FETs fabricated from CNT films with deposition times of 10, 20, 40, and 80 min are shown, their transfer characteristics are in Fig. 6. The gate current is less than 0.1 nA for all devices. The CNT FETs exhibit ambipolarity which is consistent with previously reported network CNT FETs measured in dry top gate and back gate geometry [5], [6].

The on-currents in Fig. 5b show an increase of two decades as the suspension times during device fabrication were increased from 10 min to 80 min. The increase in current is attributed to the increased number of conduction paths between source and drain electrodes as the network density increases [7], [8], [9], [10], [11]. For the CNT FETs closest to the percolation threshold (deposition times of 10 min) we see larger variation in the on and off currents as expected due to the proximity of those networks to the percolation threshold. As CNT deposition time increases we see an increase in the off current. This is because as the bundle density is increased the network nears the metallic percolation threshold, increasing the likelihood of continuous metallic segments in the network [10], [11].

The on-off ratios shown in Fig. 5c are 3 orders of magnitude higher for devices closer to percolation. More than 30% of the devices with CNT deposition times of 10 min have an on-off ratio of over 10^5^, and all are over 10^3^. The threshold voltages of the devices are shown in Fig. 5d. We observe a positive shift in *V*_*th*_ and increases in *I*_*ds*_ with increasing CNT deposition time, as expected for *p*-type CNT FETs [12], [13], [14].

### 1.5 *I*-*V* curves of pristine CNT FETs

**Fig. 7:**
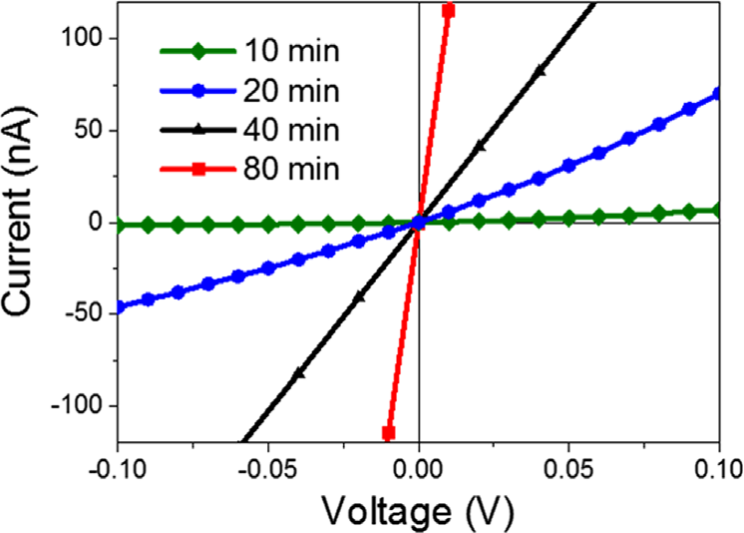
The *I*-*V* characteristics of the of the devices fabricated with CNT films on kapton substrate with deposition times of 10, 20, 40 and 80 min.

The *I*-*V* curves for pristine unfunctionalized devices show a non-linear response for devices fabricated with a CNT deposition time of 10 min and 20 min as shown in Fig. 7, while those fabricated at 40 min and 80 min CNT deposition times show a linear response. The non-linearity in the 10 min and 20 min devices indicates that the conduction is dominated by potential barriers at m-s junctions within the CNT bundle network.

## 2 Experimental design, materials, and methods

The detail experimental methods for the device fabrication, AFM measurements and electrical measurements are described in detail in the manuscript [1].
